# Segmentation of Low-Grade Brain Tumors Using Mutual Attention Multimodal MRI

**DOI:** 10.3390/s24237576

**Published:** 2024-11-27

**Authors:** Hiroyuki Seshimo, Essam A. Rashed

**Affiliations:** 1 Graduate School of Information Science, University of Hyogo, Kobe 650-0047, Japan; ad23l030@guh.u-hyogo.ac.jp; 2 Advanced Medical Engineering Research Institute, University of Hyogo, Himeji 670-0836, Japan

**Keywords:** image segmentation, low-grade brain tumor, MRI, mutual attention

## Abstract

Early detection and precise characterization of brain tumors play a crucial role in improving patient outcomes and extending survival rates. Among neuroimaging modalities, magnetic resonance imaging (MRI) is the gold standard for brain tumor diagnostics due to its ability to produce high-contrast images across a variety of sequences, each highlighting distinct tissue characteristics. This study focuses on enabling multimodal MRI sequences to advance the automatic segmentation of low-grade astrocytomas, a challenging task due to their diffuse and irregular growth patterns. A novel mutual-attention deep learning framework is proposed, which integrates complementary information from multiple MRI sequences, including T2-weighted and fluid-attenuated inversion recovery (FLAIR) sequences, to enhance the segmentation accuracy. Unlike conventional segmentation models, which treat each modality independently or simply concatenate them, our model introduces mutual attention mechanisms. This allows the network to dynamically focus on salient features across modalities by jointly learning interdependencies between imaging sequences, leading to more precise boundary delineations even in regions with subtle tumor signals. The proposed method is validated using the UCSF-PDGM dataset, which consists of 35 astrocytoma cases, presenting a realistic and clinically challenging dataset. The results demonstrate that T2w/FLAIR modalities contribute most significantly to the segmentation performance. The mutual-attention model achieves an average Dice coefficient of 0.87. This study provides an innovative pathway toward improving segmentation of low-grade tumors by enabling context-aware fusion across imaging sequences. Furthermore, the study showcases the clinical relevance of integrating AI with multimodal MRI, potentially improving non-invasive tumor characterization and guiding future research in radiological diagnostics.

## 1. Introduction

Astrocytoma is one of the most common low-grade brain tumors, with the potential to progress into higher-grade, more aggressive forms if not properly managed. The clinical treatment protocols for astrocytoma typically include tumor resection, chemotherapy, and radiotherapy, with the aim of controlling the tumor’s growth while preserving brain function [1]. Despite the relatively high survival rate in patients with low-grade astrocytomas, careful consideration of treatment protocols remains essential [2]. Accurate diagnosis and monitoring are crucial for planning treatment strategies, as brain cancer therapies need to minimize damage to healthy tissues in this highly sensitive organ. Magnetic resonance imaging (MRI) is the primary imaging modality used for both diagnosis and follow-up care, offering high-contrast, non-invasive insights into tumor size, location, and response to treatment.

Accurate tumor segmentation plays a pivotal role in clinical workflows by providing quantitative measurements for diagnosis, treatment planning, and monitoring tumor progression. However, the manual segmentation of brain tumors is a labor-intensive task that requires expert knowledge, making it prone to inter-observer variability. Furthermore, given the increasing demand for healthcare services and limited manpower in the healthcare sector, manual segmentation is not always feasible [3]. As a result, automatic and semi-automatic segmentation methods have become a priority to improve efficiency and reproducibility. Several computational approaches have been explored for the segmentation of astrocytomas. Traditional methods rely on hand-crafted features such as texture, intensity, or spatial information derived from MRI, known as conventional machine learning methods which were used for long time to solve this problem [4]. (1) Techniques like support vector machines (SVMs) and random forests (RFs) have been employed to perform segmentation. However, these methods struggle with the heterogeneity of tumor appearance and require manual feature engineering, limiting their scalability across datasets. (2) Atlas-based segmentation enables prior anatomical knowledge to guide the segmentation [5,6]. These methods align the patient’s MRI with a pre-labeled brain atlas to estimate tumor regions. Region-growing algorithms, on the other hand, segment based on pixel similarity starting from an initial seed point. While these methods perform well with well-defined tumor boundaries, they often fail in cases of diffuse low-grade astrocytomas with irregular or poorly defined margins. (3) Deep learning has emerged as the state-of-the-art approach for brain tumor segmentation. Models such as U-Net and its variants have demonstrated remarkable improvements in segmentation accuracy, particularly in challenging scenarios [7]. A self-supervised method that enables the use of Wavelet transform demonstrates an improvement in brain tumor segmentation using different MRI sequences [8]. Several reviews have summarized the research progress in this field using hybrid techniques [9] and federated learning [10]. These models can automatically learn complex, hierarchical features from large datasets, eliminating the need for manual feature extraction. However, they are highly data-dependent, and their performance can degrade when trained on limited or imbalanced datasets.

In this study, we propose a mutual-attention-based deep learning model that leverages multiple MRI sequences to address the limitations of conventional segmentation methods. The key innovation lies in the mutual attention mechanism, which enables the model to learn meaningful interactions between different modalities, such as T2-weighted and FLAIR images, enhancing its ability to detect tumor boundaries with precision. By focusing attention not only within each modality but also across modalities, the proposed model adapts to the specific challenges of low-grade astrocytoma segmentation. This approach is validated on the UCSF-PDGM dataset, demonstrating improved accuracy compared to traditional strategies. This paper is organized as follows: Section 2 provides an overview of related work on segmentation methodologies for brain tumors from MRI scans. The dataset used in this study and the proposed framework are detailed in Section 3. The results are presented in Section 4 and discussed in Section 5. The manuscript concludes with Section 6.

## 2. Related Work

Brain tumor segmentation has seen significant advancements with the adoption of diverse deep learning architectures, each contributing to improved accuracy and clinical utility. One notable approach is the dual-path network based on multi-modal feature fusion (MFF-DNet), which enhances segmentation precision by integrating multi-scale features from low-, middle-, and high-level layers. This method effectively addresses the heterogeneity of gliomas but suffers from increased computational complexity and the risk of vanishing gradients in multi-modal channels [11]. Similarly, ZNet utilizes an encoder–decoder structure with skip connections, achieving a mean Dice similarity coefficient of 0.92 for two-dimensional (2D) MRI segmentation. While ZNet demonstrates reliable performance in 2D tasks, its limited ability to handle three-dimensional (3D) data constrains its utility for volumetric MRI segmentation [12].

Multi-task learning has also emerged as a promising strategy, particularly for detecting small tumors. For example, Ngo et al. developed a network that leverages dilated convolution and multi-task learning, focusing on feature reconstruction to preserve small tumor details often lost during down-sampling. This approach shows promise in early cancer detection but struggles with computational overhead when applied to large datasets or complex 3D structures [13]. Additionally, UNet++ [14] has been introduced as an extension of the U-Net [15] architecture, employing a nested structure to mitigate overfitting and improve segmentation accuracy. Although UNet++ achieves superior performance for low-grade gliomas, it requires significantly more memory and computational resources compared to simpler architectures [16].

Further refinements in brain tumor segmentation have been pursued through cascaded architectures. A pediatric glioma segmentation pipeline, for instance, employs two consecutive U-Net models: the first for coarse localization and the second for refined segmentation. While this two-step approach achieves high accuracy and robust radiomic classification, the increased computational demand makes it less suitable for real-time clinical applications [17]. Another study consider manual attention labeling such as drawing an ellipse around the suspected region [18]. These efforts illustrate the continuous progress in developing architectures that balance precision with computational efficiency.

In recent studies, 3D fully convolutional networks (3D FCN) have been employed to capture complex spatial features in multimodal MRI scans. These models provide better volumetric segmentation but come with the trade-off of increased computational overhead and a heightened risk of overfitting without extensive datasets [19,20]. To address the challenges associated with limited datasets, transfer learning has been explored. Pre-trained models, fine-tuned for specific medical imaging tasks, show efficient performance even with smaller datasets. However, these models often require domain-specific adjustments to achieve optimal results [21]. Hybrid approaches, which combine convolutional neural networks (CNNs) with traditional machine learning models like RF, offer enhanced performance by leveraging both feature extraction and probabilistic modeling. Nonetheless, these models face challenges in interpretability and scalability, particularly when applied to large datasets or real-time clinical settings [20]. Multi-pathway architectures represent another avenue of research, focusing on extracting features at multiple scales using dilated convolutions. A brief list of deep learning methods is given in Table 1. These architectures have shown promise in segmenting tumor subregions, such as edema and necrotic tissue, by capturing both fine and coarse features. However, their computational intensity and susceptibility to vanishing gradients necessitate careful design to ensure stable training [19]. Despite these challenges, such advanced segmentation frameworks significantly enhance clinical workflows by supporting more precise diagnosis, treatment planning, and monitoring of disease progression.

Overall, the diverse range of strategies for brain tumor segmentation illustrates the field’s rapid evolution. While these approaches address various aspects of segmentation, such as heterogeneity, multi-modal fusion, and small tumor detection, they also introduce trade-offs between segmentation accuracy, computational demand, and clinical feasibility.

## 3. Materials and Methods

In the related work discussed above, several network architectures have been discussed with a variety of network designs and sequences of layers [10]. However, it has hardly focused on low-grade gliomas such as astrocytomas. This is important because low-grade tumors are more likely to be curable and patient survival rate is relatively higher than in the case of higher-grade ones [33,34]. In this study, we focus on low-grade astrocytomas wherein treatment would likely lead to higher survival rate.

### 3.1. Image Dataset

**Figure 1:**
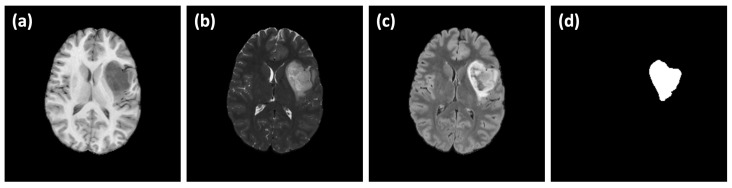
Sample of the UCSF-PDGM dataset. (**a**) T1w, (**b**) T2w and (**c**) FLAIR MRI scans with (**d**) astrocytoma segmentation label.

The UCSF-PDGM dataset provides a valuable multimodality open-source repository for brain gliomas imaging, with MRI scans from 501 patients and corresponding histopathologically confirmed grade II-IV diffuse gliomas [35]. Images are acquired using a standardized 3T MRI protocol with advanced techniques such as ASL and HARDI. Moreover, the dataset includes key genetic biomarkers like IDH mutation and MGMT promoter methylation status. More importantly, the dataset includes a comprehensive tumor segmentation verified by radiologists as well as several MRI sequences such as T1w, T2W, FLAIR, DWI, SWI, HARDI(FA), ASL and T1w Gad. This dataset is selected to be used in this study as it provides a rich and comprehensive imaging moralities and it is available as open source, which make it a valuable benchmark for validation and reproduction. A sample of the UCSF-PDGM dataset is shown in Figure 1. A subset of 35 subjects is selected wherein the diagnosis of astrocytoma (grade II) is confirmed. Image size is 240 × 240 × 155 pixels (1 mm resolution) in NIfTI formats. The dataset is divided into 5 batches (each of 7 randomly selected subjects) for 5-fold cross-validation study.

### 3.2. Network Architecture

**Figure 2:**
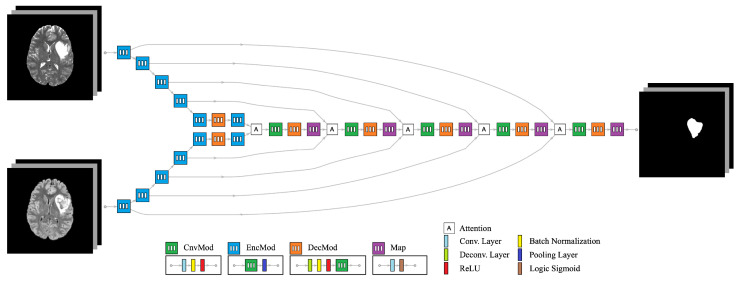
Neural network architecture for dual input mutual attention segmentation.

The proposed network architecture is an end-to-end convolutional neural network (CNN) designed to connect *I* anatomical images to *O* segmentation labels. An example configuration is illustrated in Figure 2, where I=2 and O=1. This architecture comprises several interconnected modules to efficiently extract and exchange features across different modalities:

- Convolution Module (CnvMod): This module consists of a 2D convolution layer with a kernel size of 3×3, followed by batch normalization and a Rectified Linear Unit (ReLU). It acts as the basic building block, enabling feature extraction by detecting spatial patterns within each modality.
- Encoder Module (EncMod): The encoder processes input data through a CnvMod, followed by a 2×2 max pooling layer. This module extracts high-level spatial features and reduces spatial dimensions, ensuring that the model focuses on the most salient information.
- Decoder Module (DecMod): The decoder includes a deconvolution layer to restore the spatial resolution, followed by batch normalization, ReLU activation and a CnvMod. This design allows the model to gradually reconstruct the segmentation map from the learned features, ensuring accurate boundary delineation.
- Mutual Attention Layer: This layer integrates features from multiple data streams, enabling interaction between different modalities. The mutual attention mechanism dynamically weighs the importance of features from each modality, ensuring that complementary information is captured and utilized for segmentation. It enhances the exchange of relevant features between modalities, such as T2w and FLAIR, which may highlight different tumor characteristics.
- Map Module (Map): The map module applies a final 2D convolution layer, followed by a sigmoid activation function, to generate the final segmentation output. This layer ensures that the output is normalized between 0 and 1, representing the probability of each voxel belonging to the tumor region.

The architecture leverages individual encoder tracks for each input modality, which are processed through multiple convolutional operations to extract modality-specific features. These encoder tracks are designed to work in parallel, ensuring that relevant features from different anatomical images are captured independently. The decoder tracks are tasked with generating segmentation labels through a combination of deconvolutional and convolutional operations, gradually reconstructing the output while maintaining spatial resolution.

A key strength of this design lies in the mutual attention layer, which facilitates a seamless exchange of features between the different modalities. This mechanism not only allows the model to learn modality-specific information but also strengthens the extraction of shared features by dynamically attending to the most relevant aspects of each input. For example, the model can emphasize FLAIR images to highlight edema regions and T2w images to capture the tumor core, resulting in more accurate and comprehensive segmentation.

The use of skip connections between the encoder and decoder modules further enhances performance by reintroducing high-resolution features lost during down-sampling. The mutual attention layer, combined with these skip connections, ensures that information flows effectively between all stages of the network, reducing the risk of feature degradation. This integrated design allows the model to adaptively learn from multimodal data, ensuring robust feature extraction and precise segmentation of astrocytoma boundaries.

In summary, the proposed network architecture, with its modular design and mutual attention mechanism, ensures that features are effectively extracted, exchanged and utilized from multiple input modalities. This capability makes the model well-suited to handle the challenges of multimodal MRI-based tumor segmentation, leading to more precise and clinically relevant outcomes.

### 3.3. Mutual Attention

**Figure 3:**
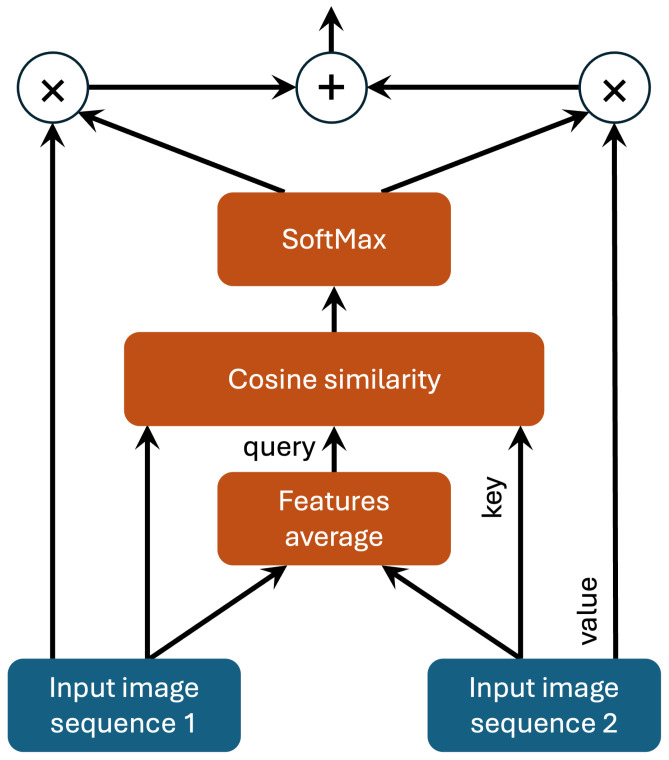
Mutual attention mechanism for two input images.

Without loss of generality, we explain the mutual attention mechanism using the simple case of two input images, with direct extensions possible for more inputs. In the proposed architecture, shown in Figure 2, two feature quantities are generated from the two input images. These feature quantities serve as inputs to the attention layer. Within the attention mechanism, an average feature quantity is computed from the two input feature quantities, serving as a shared context. The Query (Q) is extracted from this average feature quantity, while the Key (K) is extracted from each individual feature quantity. The similarity between the Query and each Key is calculated to measure how well the features align. The similarity scores are then passed through a softmax function to generate attention weights, which are multiplied by the Value (V) extracted from each feature quantity. This process produces new, weighted feature quantities. The two new feature quantities are then added together to form a final fused feature quantity, as illustrated in Figure 3. This fused representation enables the adaptive fusion of features extracted from different modalities, which can lead to improved segmentation accuracy. MRI sequences often exhibit diverse patterns of anatomical features, and the adaptive fusion of complementary information enhances generalization and provides more robust segmentation results across varying cases.

### 3.4. Pre-Processing and Normalization

**Figure 4:**
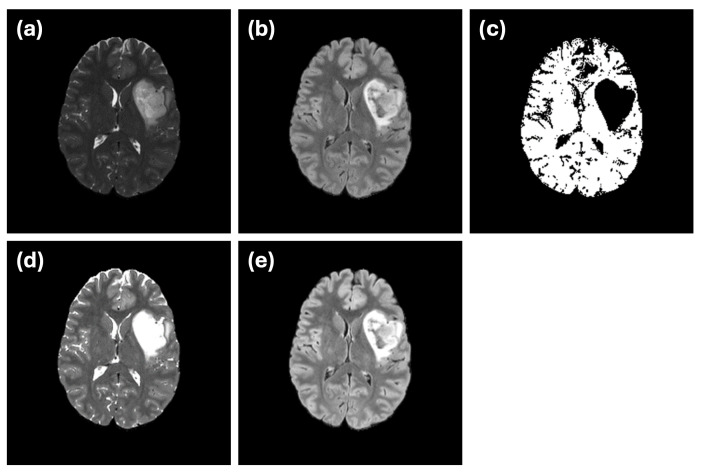
Example of parenchyama-based normalization. (**a**) T2-w and (**b**) FLAIR images are normalized to (**d**) and (**e**), respectively, using parenchyama label in (**c**).

Effective pre-processing is essential for ensuring the consistency and quality of MRI data used for training and evaluating deep learning models. The UCSF-PDGM is already pre-processed as detailed in [35]. This includes bias correction, skull stripping, resampling to unified resolution of 1 mm and image registration of different sequences. However, we found a variation in the abnormality representation in different MRI sequences, which make it difficult to use the common z-score normalization method to set image values within the range of [0, 1]. Alternatively, we normalize each subject based on image values within the parenchyma using the following formula:

(1) $$\hat{x}=\alpha +\frac{\left(x-x_{min}^{b}\right)\left(\beta -\alpha \right)}{x_{max}^{b}-x_{min}^{b}},$$

where *x* is the original image, $x_{max}^{b}$ and $x_{max}^{b}$ are the maximum and minimum values within the parenchyama, respectively. α=0.01 and β=0.99 are hyperparameters to set the bound of the normalized image. This process is illustrated in Figure 4. This normalization ensures that the input data across all modalities are on a comparable scale, preventing any single modality from dominating the learning process if pixels within abnormalities are considered.

### 3.5. Evaluation Metrics

Segmentation accuracy is evaluated using different metrics such as dice coefficient (Dc), accuracy (Ac), recall coefficient (Re), precision (Pr), interaction over union coefficient (IoU), volumetric similarity (Vs) and Hausdorff distance (Hd). These metrics are defined as follows:

(2) $$Dc=\frac{2*TP}{FP+2*TP+FN},$$

(3) $$Ac=\frac{TP+TN}{TP+FP+TN+FN},$$

(4) $$Re=\frac{TP}{TP+FN},$$

(5) $$Pr=\frac{TP}{TP+FP},$$

(6) $$IoU=\frac{TP}{TP+FP+FN},$$

(7) $$Vs=1-\frac{\left|FN-FP\right|}{2*TP+FP+FN},$$

(8) $$Hd\left(A,B\right)=\max\{\underset{a\in A}{\sup}\underset{b\in B}{\inf}|a-b|,\underset{b\in B}{\sup}\underset{a\in A}{\inf}|b-a|\},$$

where TP, TN, FP and FN represents true positive, true negative, false positive and false negative, respectively. *A* and *B* in Equation (8) represent true segmentation and model segmentation regions.

## 4. Results

The proposed model is implemented in Python on a workstation of Core i9 CPU@ 3.0 GHz, 128 GB Memory and 2 × NVIDIA RTX A4000 GPUs. The dice loss cost function and training is optimized by the Adam algorithm. Several experiments are conducted to evaluate the performance of the proposed approach.

### 4.1. Evaluation of Difference in MRI Sequences

The first experiment considers using the network architecture in Figure 2 with I=1 and O=1 (i.e., single input and single output) to evaluate the contribution of different MRI sequences in the segmentation accuracy. The purpose of this screen study is to select the most valuable modalities to be used in further investigations. We use bias-corrected T1w, T1w (with contrast), T2w, FLAIR, DWI and SWI sequences using 25 randomly selected subjects for training and the remaining 10 subjects for testing. Training is conducted using 50 epochs with batch size of 16. The results of the average dice score are listed in Table 2. These results demonstrate clear superiority in FLAIR MRI with no significant change in other modalities. Therefore, we decided to select T2w and FLAIR as potential candidates for further studies using dual modalities.

### 4.2. Performance of Mutual Attention

**Figure 5:**
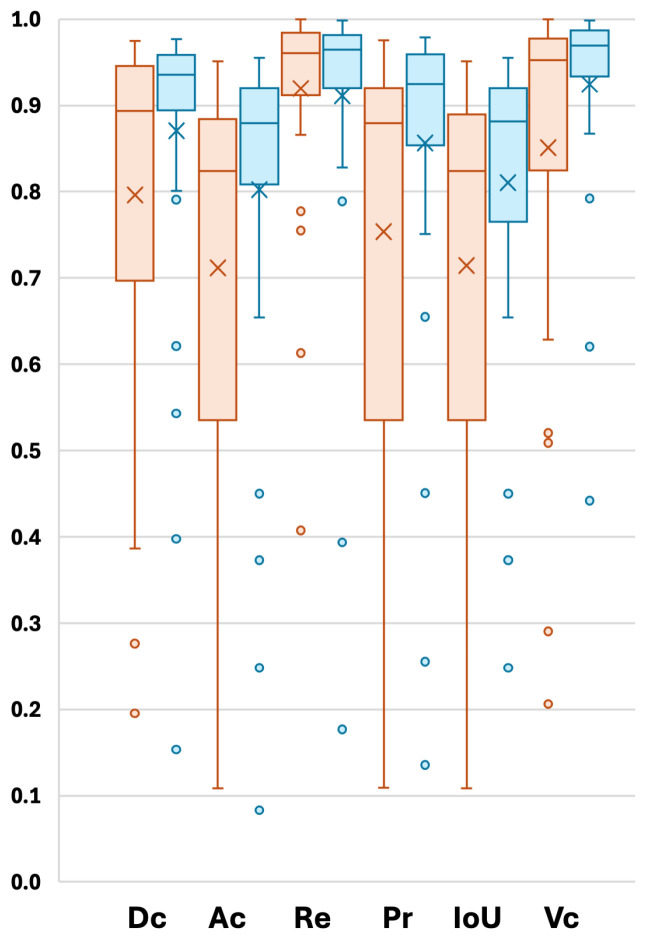
Box plot illustration for different segmentation accuracy measurements detailed in Table 3 for single modality (orange color) and dual modality (blue color).

An additional experiment was conducted using 5-fold cross-validation to evaluate the performance of the proposed model, which utilizes mutual attention between two modalities (FLAIR and T2w), in comparison with a single-modality setup using only FLAIR. The model was trained for 50 epochs with a batch size of 8. Various segmentation metrics for the five cross-validation folds are presented in Table 3, and the distribution of individual results is visualized in Figure 5. The results demonstrate that the use of mutual attention between FLAIR and T2w consistently outperforms the single-modality approach in most cases, with a notable improvement in the average Dice score, increasing from 0.7961 to 0.8707. This significant enhancement highlights the effectiveness of the proposed model in leveraging complementary information from multiple modalities to improve segmentation performance.

**Figure 6:**
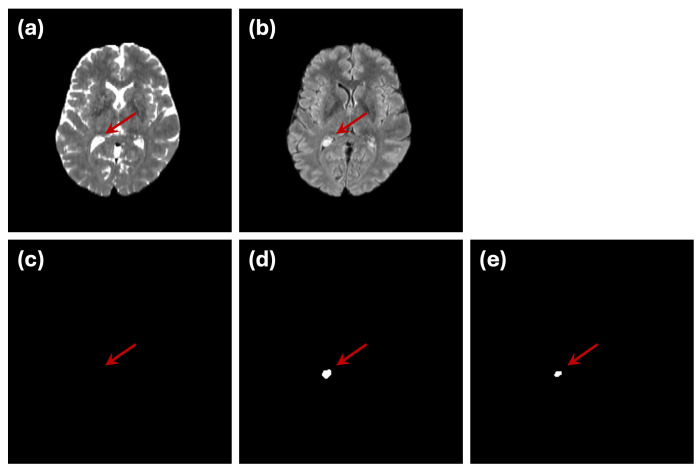
Example of a mismatch between golden truth segmentation and model segmentaion. (**a**) T2W and (**b**) FLAIR MRIs indicate a region with high brightness in lateral ventricle (labeled by red arrow) which is not represented in (**c**) golden truth segmentation but appears in different representations in (**d**) single (FLAIR) and (**e**) multi (FLAIR and T2w) modality segmentations. Due to this issue, segmentation accuracy metrics for this subject (UCSF-PDGM-0255) are remarkably low.

However, we identified one subject (subject ID: UCSF-PDGM-0255, a 40-year-old female diagnosed with a grade II astrocytoma) that exhibited poor segmentation performance in both single- and dual-modality setups. Upon further investigation and consultation with medical experts, we discovered that a hyperintense region appeared within the bilateral ventricles in the FLAIR image for this subject. Interestingly, the corresponding ground truth segmentation did not label this region as an abnormality (Figure 6), resulting in an increased rate of false positives for this case. Given the unique nature of this subject and the labeling discrepancy, we recalculated the performance metrics excluding this subject to provide a clearer assessment of the model’s typical performance. These adjusted metrics are also included in Table 3. The analysis confirms that the proposed model’s performance improves substantially when excluding this outlier case, further validating the robustness of the mutual-attention-based approach.

### 4.3. Performance of Alternative Combinations

To better understand the performance of the proposed framework with different combinations of MRI sequences, another experiment was conducted using the same setup as described in the previous section. We evaluated various combinations of T1w, T1c, T2w and FLAIR sequences, and the results were validated using Dc, Ac and Re, as shown in Table 4. The findings indicate that the T2w/FLAIR and T1c/FLAIR combinations are the most effective, achieving average Dc values of 0.8707 and 0.8656, respectively. In contrast, the T1w/T2w and T1c/T2w combinations yielded lower accuracies, with average Dc values of 0.7835 and 0.7727, respectively. Notably, the T1w/FLAIR and T1w/T1c combinations demonstrated significantly lower performance, with Dc values of 0.4751 and 0.0271, respectively. These results highlight the critical role of complementary information in achieving high segmentation performance. The superior performance of the T2w/FLAIR and T1c/FLAIR combinations underscores the importance of integrating sequences that provide distinct yet synergistic insights into anatomical and pathological features. T2w and FLAIR are particularly sensitive to fluid content and edema, while T1c enhances contrast in regions with disrupted blood–brain barriers, making these combinations effective for capturing both structural and pathological details. In contrast, the moderate performance of T1w/T2w and T1c/T2w combinations may reflect a degree of redundancy in the information provided by these sequences, limiting their complementary value. The low performance of T1w/FLAIR and T1w/T1c combinations suggests that these pairings fail to capture sufficient complementary information to enhance segmentation accuracy. These findings emphasize the need to carefully select and combine MRI sequences that contribute unique and relevant features for segmentation tasks, as well as the potential value of refining pre-processing steps or model architectures to better leverage under-performing combinations.

## 5. Discussion

### 5.1. Performance Evaluation

The results presented in this study demonstrate the effectiveness of the proposed mutual-attention-based deep learning model for astrocytoma segmentation using multimodal MRI scans. Through the integration of FLAIR and T2-weighted (T2w) images, the model fusion offers complementary information from both modalities, leading to significant improvements in segmentation performance. This enhancement underscores the value of multimodal fusion in medical imaging, wherein subtle and complex features are better captured through the interaction between multiple data streams. The incorporation of mutual attention is a key innovation of the proposed approach, enabling the model to learn meaningful interactions between modalities. By dynamically attending to the most relevant features across both FLAIR and T2w inputs, the model mitigates the limitations of single-modality segmentation, such as false positives and incomplete boundary detection. This mechanism allows for better identification of tumor regions, especially at the boundaries, where subtle variations between modalities can provide crucial information.

While the overall performance of the model is promising, one outlier case (subject ID: UCSF-PDGM-0255) highlighted the challenges of working with medical image datasets. In this case, a hyperintense region within the bilateral ventricles was visible in the FLAIR modality but was not included in the ground truth segmentation labels. Medical experts need to explore additional MRI sequences such as T1w, T1w (contrast), DWI, ADC, ASl and SWI to explore the possibility that this hypertension region is likely to be a xanthogranuloma (benign tumor of the choroid plexus). This discrepancy emphasizes the importance of accurate and consistent labeling in medical imaging datasets, as incorrect or incomplete annotations can mislead model training and evaluation. Further investigation and consultation with medical experts confirmed the difficulty of distinguishing this region, illustrating the complexity of generating reliable ground truth labels for medical image segmentation tasks. Moreover, it highlights that further investigation on models that can fusion additional MRI sequences is required to reduce false positive cases.

### 5.2. Accuracy of Golden Truth Labels

One noteworthy observation from the results presented in this study is the quality and reliability of the ground truth segmentation labels. Although the original labels were automatically generated and subsequently corrected by a group of experts [35], ensuring accurate segmentation labels remains a challenging task. This challenge arises from various sources of bias, including human error, noise interference and other confounding factors [36,37,38]. Inaccurate labels can adversely affect the training of deep learning models, leading to suboptimal performance [39].

**Figure 7:**
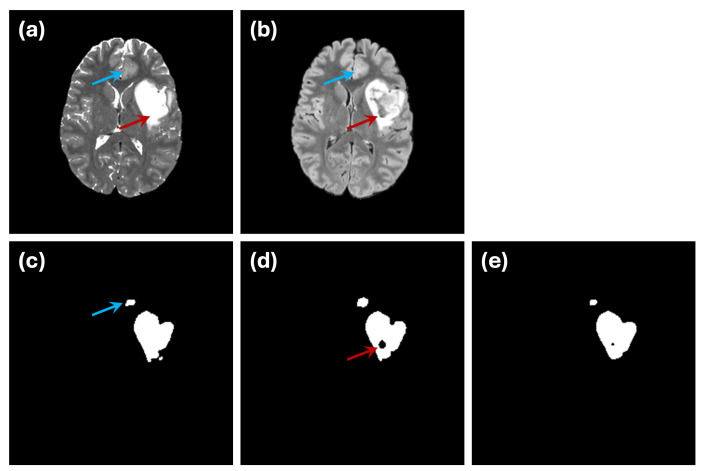
Example of a potential CSF region inside an astrocytoma. In (**a**) T2w MRI, it is challenging to distinguish CSF, as it appears within the same grayscale range as the tumor. However, it is more clearly visible in (**b**) the FLAIR image. Images (**c**–**e**) show the ground truth segmentation, single-modality (FLAIR) and dual-modality segmentations, respectively. The ground truth segmentation considers the entire region as abnormal, while the single-modality segmentation reveals a hole (indicated by the red arrow), highlighting the confusion caused by the lack of data integrity. A small abnormality region (indicated by the blue arrow) appears in normal grayscale in both MRIs, which make it difficult to be recognized accurately during training.

An example of this issue is illustrated in Figure 7. In the ground truth segmentation (Figure 7c), the abnormal region is labeled as a continuous structure, with no internal holes. However, when segmentation is performed using only the FLAIR modality (Figure 7d), an internal hole appears, likely representing a cerebrospinal fluid (CSF) region. This discrepancy highlights how single-modality input can introduce confusion due to limited data integrity. The use of dual-modality segmentation (Figure 7e) mitigates this issue by reducing the occurrence of such internal holes, illustrating the importance of incorporating complementary imaging information.

Another challenge is demonstrated by the small abnormality indicated by the blue arrow in Figure 7. While the ground truth labels mark this region as an abnormality (Figure 7c), it is difficult to discern either of the individual modalities (Figure 7a,b). This example underscores the inherent difficulty in ensuring that ground truth annotations used for training or evaluation fully and accurately represent the underlying abnormalities.

These challenges emphasize the complexities involved in creating reliable ground truth labels for medical image segmentation. Given the potential impact of inaccurate labels on model performance, further efforts are required to improve the validation and confirmation processes for these annotations. Reliable evaluation frameworks are essential to ensure that the ground truth used in deep learning tasks aligns with clinical reality, ultimately improving the robustness and applicability of segmentation models.

### 5.3. Limitations

While the proposed mutual attention-based deep learning model demonstrates promising results, several limitations must be acknowledged. One major constraint lies in the size of the dataset used in this study, which comprises only 35 subjects. This limited sample size restricts the model’s ability to generalize across a broader population and capture the full variability present in clinical settings. A larger and more diverse dataset would be necessary to ensure the robustness of the model in real-world applications. Another limitation is the reliance on only two imaging modalities, T2-weighted and FLAIR MRI, for segmentation. Although these modalities provide valuable complementary information, the inclusion of additional modalities such as T1-weighted, contrast-enhanced T1 or diffusion-weighted imaging could further improve segmentation accuracy and enhance the model’s ability to identify more complex tumor features. Future work should explore the integration of multiple imaging sequences to better capture the heterogeneity of brain tumors and improve the precision of tumor boundary delineation. Addressing these limitations in future studies would enhance the model’s generalizability and clinical relevance, paving the way for more comprehensive and reliable brain tumor segmentation frameworks.

## 6. Conclusions

In this study, we present a novel deep learning model for the segmentation of astrocytomas from multimodal MRI scans, introducing a mutual attention-based mechanism to enhance segmentation performance. Unlike conventional approaches that rely on a single modality input, our model leverages multiple modalities, focusing on meaningful interactions between T2w and FLAIR images. This innovative use of mutual attention allows the model to capture complementary information across modalities, improving the detection and delineation of tumor boundaries with enhanced precision. The originality of the proposed approach lies in the ability of the mutual attention mechanism to adaptively weigh the importance of features from different modalities. This not only improves the segmentation performance but also addresses some challenges inherent in astrocytoma segmentation, such as the subtle differences between healthy tissues and tumor boundaries. Additionally, the use of cross-validation ensures the robustness of the model evaluation, demonstrating that our method generalizes well within the constraints of the publicly available dataset. However, as with any deep learning model, challenges remain. While the results show a significant improvement over conventional single-modality approaches, further refinement is necessary to address the accurate segmentation of small tumors. This is particularly important in clinical practice, as early detection and precise delineation of small lesions are crucial for effective treatment. Future work will focus on enhancing the model’s ability to capture fine-grained tumor details, possibly through the incorporation of additional MRI sequences and the integration of advanced data augmentation strategies. Expanding the dataset to include a larger and more diverse set of samples will also be prioritized to improve the generalizability and robustness of the model in real-world clinical scenarios.

## Figures and Tables

**Table 1:** Comparison of selected deep learning methods for brain tumor segmentation.

Ref.	Year	Tumor Type	Network Architecture	MRI Sequences	Subjects/Dataset	Dc
[22]	2016	LGG, HGG	Multilayer CNN	T1w, T1c, T2w, FLAIR	BraTS2013 [23]	0.88, 0.83, 0.77 *
					BraTS2015 [24]	0.78, 0.65, 0.75 *
[25]	2017	LGG, HGG	InputCascadeCNN	T1w, T1c, T2w, FLAIR	BraTS2013	0.88, 0.79, 0.73 *
[26]	2018	LGG, HGG	2CNet, 3CNet, EnsembleNet	T1c, T2w, FLAIR	BraTS2017 [27]	0.88
[28]	2019	Stages II-IV	FCNN	FLAIR	11	0.8
[16]	2020	LGG	U-Net	FLAIR	3929	0.8521
			U-Net++			0.8910
[29]	2021	pHGG	3D U-Net	T1c, FLAIR	794	0.724
[30]	2021	LGG, HGG	MU-Net	T1w, T1c, T2w, FLAIR	BraTS2015	0.82, 0.68, 0.61 *
[17]	2024	pLGG	2-step U-Net	FLAIR	288	0.795
[31]	2024	LGG, HGG	SDV-TUNet	T1w, T1c, T2w, FLAIR	BraTS2020 [27]	0.9022, 0.8920, 0.8248 *
					BraTS2021 [27]	0.9310, 0.9099, 0.8764 *
[32]	2025	LGG, HGG	CFNet	T1w, T1c, T2w, FLAIR	BraTS2019 [27]	0.9089, 0.9052, 0.9061 *
					BraTS2020	0.9160, 0.9046, 0.9029 *

LGG: *Low-Grade Glioma*; HGG: *High-Grade Glioma*; pLGG: *Pediatric Low-Grade Glioma*; pHGG: *Pediatric High-Grade Glioma*; * *For the complete, core, and enhancing tumor regions, respectively.*

**Table 2:** Evaluation of segmentation accuracy using single MRI modality. Bold indicates the top two values.

Modality	Dc
T1w	0.6647
T1w (contrast)	0.6648
T2w	**0.6789**
FLAIR	**0.9100**
DWI	0.6647
SWI	0.6648

**Table 3:** Evaluation of segmentation accuracy using single modality (FLAIR) and dual modality (FLAIR + T2w). Bold indicates the top values.

Fold	Modality	Dc	Ac	Re	Pr	IoU	Vc	Hd
Fold-1	Single	0.8569	0.8645	0.9562	0.9006	0.8645	0.9672	38.05
	Dual	0.9472	0.9012	0.9806	0.9178	0.9012	0.9667	52.46
Fold-2	Single	0.8383	0.7374	0.9188	0.8019	0.7374	0.8819	44.30
	Dual	0.9033	0.8309	0.9346	0.8835	0.8309	0.9493	66.77
Fold-3	Single	0.8631	0.7964	0.8899	0.8316	0.8128	0.9131	28.48
	Dual	0.8409	0.7964	0.8435	0.8391	0.8377	0.9744	39.45
Fold-4	Single	0.6778	0.5584	0.9740	0.5638	0.5584	0.6919	34.45
	Dual	0.8665	0.7775	0.9765	0.7926	0.7898	0.8884	69.31
Fold-5	Single	0.7444	0.6573	0.8539	0.7329	0.6573	0.8494	41.50
	Dual	0.7959	0.7061	0.8286	0.8427	0.7061	0.8416	40.60
Average	Single	0.7961	0.7234	**0.9185**	0.7662	0.7261	0.8607	**37.35**
	Dual	**0.8707**	**0.8024**	0.9127	**0.8552**	**0.8131**	**0.9241**	53.71
Average *	Single	0.7827	0.7082	0.9243	0.7531	0.7087	0.8502	**39.13**
	Dual	**0.8767**	**0.8036**	**0.9266**	**0.8583**	**0.8082**	**0.9140**	56.57

* *Excluding subject ID: UCSF-PDGM-0255.*

**Table 4:** Evaluation of segmentation accuracy using different combination of MRI sequences. Bold indicates the top values.

	Dc			Ac			Re		
	**T1c**	**T2w**	**FLAIR**	**T1c**	**T2w**	**FLAIR**	**T1c**	**T2w**	**FLAIR**
**T1w**	0.0271	0.7835	0.4751	0.0140	0.6913	0.3761	0.0155	0.8911	0.4208
**T1c**		0.7727	0.8656		0.6771	0.7836		0.9052	**0.9138**
**T2w**			**0.8707**			**0.8024**			0.9127

## Data Availability

The data used in this study are available from https://www.cancerimagingarchive.net/collection/ucsf-pdgm/ (accessed on 9 July 2024).
